# Sodium potassium hydrogen citrate, NaKHC_6_H_5_O_7_

**DOI:** 10.1107/S2056989016000232

**Published:** 2016-01-13

**Authors:** Alagappa Rammohan, James A. Kaduk

**Affiliations:** a Atlantic International University, Honolulu HI , USA; b Illinois Institute of Technology, 3101 S. Dearborn St., Chicago IL 60616 , USA

**Keywords:** powder diffraction, density functional theory, citrate, sodium, potassium, crystal structure

## Abstract

The crystal structure of sodium potassium hydrogen citrate has been solved and refined using laboratory X-ray powder diffraction data, and optimized using density functional theory techniques. The Na^+^ cation is six-coordinate, with a bond-valence sum of 1.17. The K^+^ cation is also six-coordinate, with a bond-valence sum of 1.08. The distorted [NaO_6_] octahedra share edges, forming chains along the *a* axis. The likewise distorted [KO_6_] octahedra share edges with the [NaO_6_] octahedra on either side of the chain, and share corners with other [KO_6_] octahedra, resulting in triple chains along the *a* axis. The most prominent feature of the structure is the chain along [111] of very short, very strong hydrogen bonds; the O⋯O distances are 2.414 and 2.400 Å. The Mulliken overlap populations in these hydrogen bonds are 0.138 and 0.142 e, which correspond to hydrogen-bond energies of 20.3 and 20.6 kcal mol^−1^.

## Abstract 2

The crystal structure of sodium potassium hydrogen citrate has been solved and refined using laboratory X-ray powder diffraction data, and optimized using density functional techniques. The most prominent feature of the structure is the chain along [111] of very short, very strong hydrogen bonds; the O⋯O distances are 2.414 and 2.400 Å.

## Chemical context   

**Figure 1:**
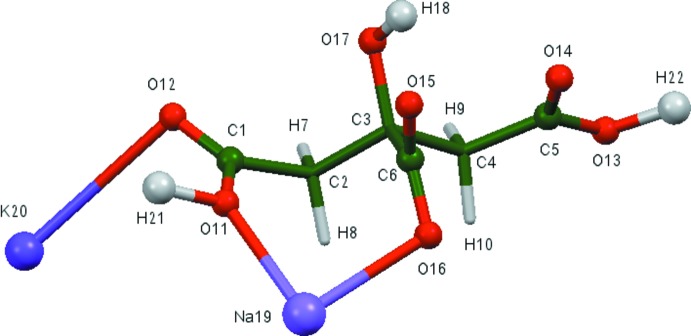
The asymmetric unit, with the atom numbering and 50% probability spheroids.

We have carried out a systematic study of the crystal structures of Group 1 (alkali metal) citrate salts to understand the anion’s conformational flexibility, ionization, coordination tendencies, and hydrogen bonding. Most of the new structures were solved using powder diffraction data (laboratory and/or synchrotron), but single crystals were used where available. The general trends and conclusions about the 16 new compounds and 12 previously characterized structures are being reported separately (Rammohan & Kaduk, 2015 ▸). The initial study considered salts containing one type of Group 1 cation. The title compound (Fig. 1 ▸) represents the beginning of an extension of the study to salts containing more than one alkali metal cation.
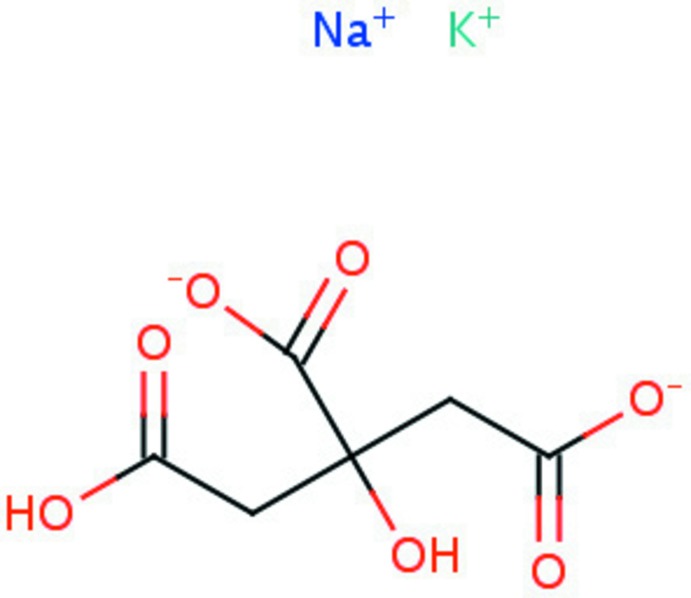


## Structural commentary   

**Figure 2:**
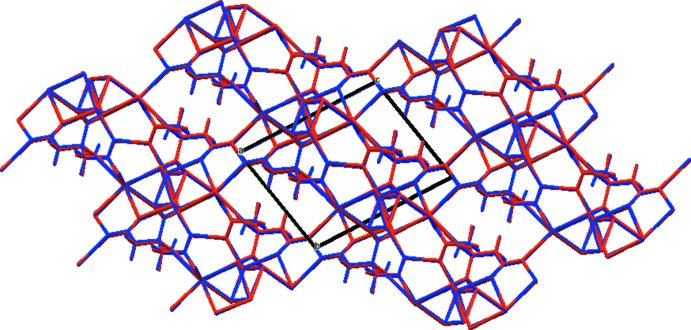
Comparison of the refined and optimized structures of sodium potassium hydrogen citrate. The refined structure is in red, and the DFT-optimized structure is in blue.

The root-mean-square deviation of the non-hydrogen atoms in the refined and optimized structures is only 0.088 Å. A comparison of the refined and optimized structures is given in Fig. 2 ▸. The excellent agreement between the structures is strong evidence that the experimental structure is correct (van de Streek & Neumann, 2014 ▸). This discussion uses the DFT-optimized structure. Most of the bond lengths, and all of the bond angles and torsion angles fall within the normal ranges indicated by a *Mercury* Mogul Geometry Check (Macrae *et al.*, 2008 ▸). Only the C6—O15 [observed = 1.281 (4), optimized = 1.268, normal = 1.20 (2) Å, Z-score = 2.7] and C1—O11 [observed = 1.260 (4), optimized = 1.318, normal = 1.330 (3) Å, Z-score = 3.9] bonds are flagged as unusual. The citrate anion occurs in the *trans*,*trans*-conformation (about C2—C3 and C3—C4), which is one of the two low-energy conformations of an isolated citrate. The central carboxyl­ate group and the hydroxyl group occur in the normal planar arrangement. The citrate chelates to Na19 through the terminal carboxyl­ate oxygen O11 and the central carboxyl­ate oxygen O16. The Na^+^ cation is six-coordinate, with a bond-valence sum of 1.17. The K^+^ cation is also six-coordinate, with a bond-valence sum of 1.08. Both cations are thus slightly crowded. The metal–oxygen bonding is ionic, based on the Mulliken overlap populations.

The Bravais–Friedel–Donnay–Harker (Bravais, 1866 ▸; Friedel, 1907 ▸; Donnay & Harker, 1937 ▸) morphology suggests that we might expect platy morphology for sodium potassium hydrogen citrate, with {001} as the principal faces. A 4th-order spherical harmonic preferred orientation model was included in the refinement; the texture index was only 1.013, indicating that preferred orientation was not significant in this rotated flat-plate specimen. The powder pattern is included in the Powder Diffraction File as entry 00-065-1255.

## Supra­molecular features   

**Figure 3:**
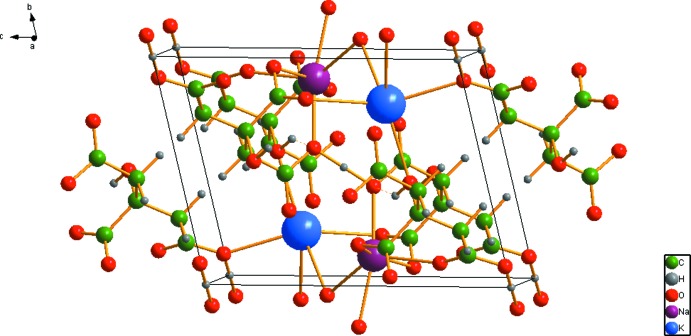
Crystal structure of NaKHC_6_H_5_O_7_, viewed approximately down the *a* axis.

In the crystal structure (Fig. 3 ▸), distorted [NaO_6_] octahedra share edges to form chains along the *a* axis. The likewise distorted [KO_6_] octahedra share edges with the [NaO_6_] octahedra on either side of the chain, and share corners with other [KO_6_] octahedra, resulting in triple chains along the *a* axis. The most prominent feature of the structure is the chain along [111] of very short, very strong O—H⋯O hydrogen bonds (Table 1 ▸); the refined O⋯O distances are 2.385 (15) and 2.346 (14) Å, and the optimized O⋯O distances are 2.414 and 2.400 Å. The Mulliken overlap populations in these hydrogen bonds are 0.138 and 0.142 e, which correspond to hydrogen bond energies of 20.3 and 20.6 kcal mol^−1^. The distances indicate that these are among the shortest O—H⋯O hydrogen bonds ever reported. H18 forms bifurcated hydrogen bonds; one is intra­molecular to O15, and the other inter­molecular to O11.

## Database survey   

Details of the comprehensive literature search for citrate structures are presented in Rammohan & Kaduk (2015 ▸). A reduced cell search in the Cambridge Structural Database (Groom & Allen, 2014 ▸) (increasing the default tolerance from 1.5 to 2.0%, to account for the differences between ambient and low-temperature lattice parameters) yielded 35 hits, but limiting the chemistry to C, H, O, Na, and K only resulted in no hits. The powder pattern matched no entry in the Powder Diffraction File (ICDD, 2015 ▸).

## Synthesis and crystallization   

2.0832 g (10.0 mmol) H_3_C_6_H_5_O_7_(H_2_O) was dissolved in 10 mL deionized water. 0.5282 g Na_2_CO_3_ (10.0 mmol Na, Sigma–Aldrich) and 0.6913 g K_2_CO_3_ (10.0 mmol, Sigma–Aldrich) were added to the citric acid solution slowly with stirring. The resulting clear colourless solution was evaporated to dryness in a 393 K oven.

## Refinement details   

**Figure 4:**
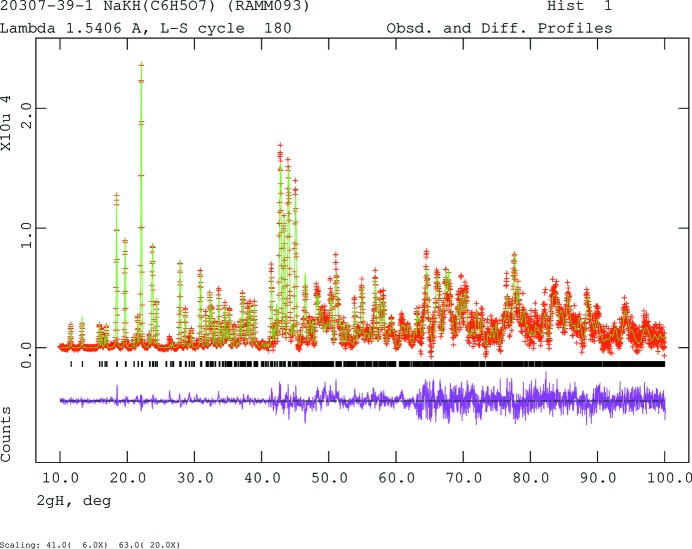
Rietveld plot for the refinement of NaKHC_6_H_5_O_7_. The red crosses represent the observed data points, and the green line is the calculated pattern. The magenta curve is the difference pattern, plotted at the same scale as the other patterns. The vertical scale has been multiplied by a factor of 6 for 2θ > 41.0°, and by a factor of 20 for 2θ > 63.0°. The row of black tick marks indicates the reflection positions for the phase.

The powder pattern (Fig. 4 ▸) was indexed using *Jade 9.5* (MDI, 2012 ▸). Pseudovoigt profile coefficients were as parameterized in Thompson *et al.* (1987 ▸) and the asymmetry correction of Finger *et al.* (1994 ▸) was applied and microstrain broadening by Stephens (1999 ▸). The structure was solved with *FOX* (Favre-Nicolin & Černý, 2002 ▸) using a citrate, Na, and K as fragments. Two of the 10 solutions yielded much lower cost functions than the others. Centrosymmetric pairs of close O⋯O contacts made it clear that H21 and H22 were located on centers of symmetry between these oxygen atoms, forming very strong hydrogen bonds. The hydrogen atoms were included at fixed positions, which were re-calculated during the course of the refinement. Crystal data, data collection and structure refinement details are summarized in Table 2 ▸. The *U*_iso_ of C2, C3, and C4 were constrained to be equal, and those of H7, H8, H9, and H10 were constrained to be 1.3× that of these carbon atoms. The *U*_iso_ of C1, C5, C6, and the oxygen atoms were constrained to be equal, and that of H18 was constrained to be 1.3× this value. The *U*_iso_ of H21 and H22 were fixed.

### Density functional geometry optimization   

A density functional geometry optimization (fixed experimental unit cell) was carried out using *CRYSTAL09* (Dovesi *et al.*, 2005 ▸). The basis sets for the H, C, and O atoms were those of Gatti *et al.* (1994 ▸), the basis sets for Na and K were those of Dovesi *et al.* (1991 ▸). The calculation used 8 k-points and the B3LYP functional, and took about 42 h on a 2.8 GHz PC. The observed *U*_iso_ were assigned to the refined values.

## Supplementary Material

Crystal structure: contains datablock(s) RAMM093_publ, ramm093_DFT. DOI: 10.1107/S2056989016000232/br2256sup1.cif

Structure factors: contains datablock(s) RAMM093_publ. DOI: 10.1107/S2056989016000232/br2256RAMM093_publsup2.hkl

Rietveld powder data: contains datablock(s) RAMM093_publ. DOI: 10.1107/S2056989016000232/br2256RAMM093_publsup3.rtv

CCDC references: 1445596, 1445595

Additional supporting information:  crystallographic information; 3D view; checkCIF report

## Figures and Tables

**Table 1:** Hydrogen-bond geometry (Å, °, e)

*D*—H⋯*A*	*D*—H	H⋯*A*	*D*⋯*A*	*D*—H⋯*A*	Overlap
O11—H21⋯O11^i^	1.207	1.207	2.414	180.0	0.138
O13—H22⋯O13^ii^	1.200	1.200	2.400	180.0	0.142
O17—H18⋯O15	0.971	2.179	2.676	110.3	0.033
O17—H18⋯O11^iii^	0.971	2.227	3.060	143.1	0.028

Symmetry codes: (i) 1 − *x*, 1 − *y*, 1 − *z*; (ii) 2 − *x*, 2 − *y*, 2 − *z*; (iii) 1 + *x*, *y*, *z*.

**Table 2:** Experimental details

	Powder data
Crystal data	
Chemical formula	NaK(C_6_H_6_O_7_)
*M*_r_	252.19
Crystal system, space group	Triclinic, *P* 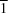
Temperature (K)	300
*a*, *b*, *c* (Å)	5.99933 (18), 8.2277 (2), 10.1419 (3)
α, β, γ (°)	74.8964 (19), 76.019 (2), 71.4496 (14)
*V* (Å^3^)	451.27 (3)
*Z*	2
Radiation type	*K*α_1_, *K*α_2_, λ = 1.540629, 1.544451 Å
Specimen shape, size (mm)	Flat sheet, 24 × 24

Data collection	
Diffractometer	Bruker D2 Phaser
Specimen mounting	Standard holder
Data collection mode	Reflection
Data collection method	Step
θ values (°)	2θ_min_ = 4.908 2θ_max_ = 99.914 2θ_step_ = 0.020

Refinement	
*R* factors and goodness of fit	*R*_p_ = 0.034, *R*_wp_ = 0.046, *R*_exp_ = 0.024, *R*(*F* ^2^) = 0.08172, χ^2^ = 4.040
No. of data points	4452
No. of parameters	98
No. of restraints	29
H-atom treatment	Only H-atom displacement parameters refined

The same symmetry and lattice parameters were used for the DFT calculation. Computer programs: *DIFFRAC.Measurement* (Bruker, 2009 ▸), *PowDLL* (Kourkoumelis, 2013 ▸), *FOX* (Favre-Nicolin & Černý, 2002 ▸), *GSAS* (Larson & Von Dreele, 2004 ▸), *EXPGUI* (Toby, 2001 ▸), *DIAMOND* (Putz & Brandenburg, 2015 ▸), *publCIF* (Westrip, 2010 ▸).

## supplementary crystallographic information

### Crystal data

**Table:**

NaKHC_6_H_5_O_7_	*c* = 10.1419 Å
*M**_r_* = 252.17	α = 74.8964°
Triclinic, *P*1	β = 76.0187°
Hall symbol: -P 1	γ = 71.4496°
*a* = 5.9993 Å	*V* = 451.27 Å^3^
*b* = 8.2277 Å	*Z* = 2

### Data collection

**Table:**

Density functional calculation	

### Fractional atomic coordinates and isotropic or equivalent isotropic displacement parameters (Å^2^)

**Table:**

	*x*	*y*	*z*	*U*_iso_*/*U*_eq_	
C1	0.58473	0.47461	0.69008	0.02150*	
C2	0.58840	0.56265	0.80315	0.00260*	
C3	0.77491	0.66691	0.77024	0.00260*	
C4	0.74288	0.74284	0.89935	0.00260*	
C5	0.90458	0.85546	0.89044	0.02150*	
C6	0.74020	0.81902	0.64154	0.02150*	
H7	0.62719	0.46044	0.89439	0.00340*	
H8	0.41202	0.65122	0.82730	0.00340*	
H9	0.77282	0.63557	0.98912	0.00340*	
H10	0.55890	0.82308	0.92211	0.00340*	
O11	0.49077	0.58291	0.58433	0.02150*	
O12	0.65591	0.31455	0.69939	0.02150*	
O13	0.88392	0.90257	1.00623	0.02150*	
O14	1.04072	0.89738	0.78176	0.02150*	
O15	0.90892	0.81600	0.53821	0.02150*	
O16	0.54704	0.93850	0.64884	0.02150*	
O17	1.00409	0.54419	0.74830	0.02150*	
H18	1.10980	0.60623	0.68105	0.02790*	
Na19	0.26024	0.87640	0.55043	0.05110*	
K20	0.17511	0.20585	0.71480	0.04060*	
H21	0.50000	0.50000	0.50000	0.03000*	
H22	1.00000	1.00000	1.00000	0.03000*	

### Bond lengths (Å)

**Table:**

C1—C2	1.515	C4—H10	1.095
C1—O11	1.318	C5—O13	1.297
C1—O12	1.234	C5—O14	1.244
C2—C3	1.543	C6—O15	1.268
C2—H7	1.092	C6—O16	1.259
C2—H8	1.090	O11—H21	1.207
C3—C4	1.540	O13—H22	1.200
C3—C6	1.558	O17—H18	0.971
C3—O17	1.430	H21—O11^i^	1.207
C4—C5	1.515	H22—O13^ii^	1.200
C4—H9	1.095		

Symmetry codes: (i) −*x*+1, −*y*+1, −*z*+1; (ii) −*x*+2, −*y*+2, −*z*+2.

### Hydrogen-bond geometry (Å, º)

**Table:**

*D*—H···*A*	*D*—H	H···*A*	*D*···*A*	*D*—H···*A*
O13—H22···O13	1.200	1.200	2.400	180.0
O11—H21···O11	1.207	1.207	2.414	180.0
O17—H18···O15	0.971	2.179	2.676	110.3
O17—H18···O11	0.971	2.227	3.060	143.1
